# Air-sea disequilibrium enhances ocean carbon storage during glacial periods

**DOI:** 10.1126/sciadv.aaw4981

**Published:** 2019-06-12

**Authors:** S. Khatiwala, A. Schmittner, J. Muglia

**Affiliations:** 1 Department of Earth Sciences, University of Oxford, Oxford, UK.; 2 College of Earth, Ocean, and Atmospheric Sciences, Oregon State University, Corvallis, OR, USA.

## Abstract

The prevailing hypothesis for lower atmospheric carbon dioxide (CO_2_) concentrations during glacial periods is an increased efficiency of the ocean’s biological pump. However, tests of this and other hypotheses have been hampered by the difficulty to accurately quantify ocean carbon components. Here, we use an observationally constrained earth system model to precisely quantify these components and the role that different processes play in simulated glacial-interglacial CO_2_ variations. We find that air-sea disequilibrium greatly amplifies the effects of cooler temperatures and iron fertilization on glacial ocean carbon storage even as the efficiency of the soft-tissue biological pump decreases. These two processes, which have previously been regarded as minor, explain most of our simulated glacial CO_2_ drawdown, while ocean circulation and sea ice extent, hitherto considered dominant, emerge as relatively small contributors.

## Teaser

Temperature and iron fertilization are more important in driving glacial-interglacial CO_2_ cycles than previously thought.

## INTRODUCTION

**Fig. 1:**
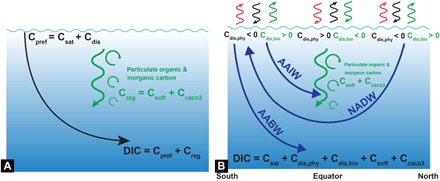
Schematic of ocean carbon decomposition. (**A**) The concentration of DIC in the ocean interior is determined by surface (“preformed”) carbon (C_pref_) transported passively by ocean circulation and regenerated carbon (C_reg_ = C_soft_ + C_caco3_) that has accumulated in a water parcel since it was last at the surface. Typically, the efficiency of the biological pump is measured as the total amount of C_reg_ in the ocean, which is dominated by C_soft_. (**B**) However, biology (shown in green) also affects surface DIC by limited outgassing of upwelling regenerated carbon at high latitudes, which increases C_pref_ over its equilibrium value (C_sat_) to create a positive disequilibrium (C_dis,bio_). Likewise, carbon removal from the surface through photosynthesis and slow ingassing creates a negative C_dis,bio_ in low-latitude oligotrophic regions of the ocean. Physical processes (black) such as surface heat fluxes (red) similarly lead to disequilibrium (C_dis,phy_). North Atlantic Deep Water (NADW) is relatively well equilibrated with the atmosphere because of its long surface exposure before sinking, whereas Antarctic Bottom Water (AABW) and Antarctic Intermediate Water (AAIW) exhibit larger disequilibria due to short surface exposure before sinking.

The ocean is the largest reservoir of carbon readily exchangeable with the atmosphere on millennial time scales. This is a consequence of both the carbonate chemistry, which makes CO_2_ highly soluble in seawater, creating a large dissolved inorganic carbon (DIC) pool, and physical and biological processes that transport carbon from the surface to the deep ocean (*1*). Air-sea gas exchange and the temperature-dependent solubility of CO_2_ concentrate carbon in the cold polar waters that fill the deep ocean, giving them a high “preformed” (C_pref_) DIC concentration (Fig. 1), a process known as the “solubility pump” (*2*). The “biological pump” further intensifies subsurface storage through the sinking and regeneration of biologically fixed particulate organic (C_soft_; “soft-tissue”) and inorganic (C_caco3_; calcium carbonate or “hard tissue”) carbon, subject to the rate at which ocean circulation and air-sea exchange re-equilibrate the dissolved carbon with the atmosphere.

A number of different mechanisms involving changes in these “pumps” (*3*) have been proposed to explain the observed ~90 parts per million (ppm) glacial atmospheric CO_2_ ($p{\text{CO}}_{2}^{\text{atm}}$) drawdown. While cooler ocean temperatures should lead to higher concentrations of dissolved CO_2_ in the glacial ocean, this effect, quantified using box models and assuming that CO_2_ is in equilibrium between the ocean and atmosphere, has typically either been regarded as a minor contribution (16 to 30 ppm) to the full glacial-interglacial difference in CO_2_ (*1*, *4*–*6*) or ignored altogether (*3*, *7*). Most theories have therefore invoked a glacial increase in the efficiency of the soft-tissue biological pump, i.e., an increase in C_soft_. One proposed mechanism for this is through a more sluggish, stratified, and isolated glacial deep ocean where C_soft_ can accumulate (*3*, *4*, *6*–*11*). This idea is supported by recent radiocarbon reconstructions indicating that whole deep ocean ^14^C ages, a measure of the time of separation from the atmosphere, during the Last Glacial Maximum [LGM; ~19 thousand years before present (ka BP)] were several hundred years older than during the Holocene. Assuming air-sea equilibrium or a fixed relationship between radiocarbon and respired carbon, this apparent aging has been estimated to explain as much as half (*11*) or more (*9*) of the glacial-interglacial CO_2_ change. Another process that would increase C_soft_ is enhanced biological productivity due to increased iron input via dust deposition (*12*). However, the efficacy of “iron fertilization” in lowering $p{\text{CO}}_{2}^{\text{atm}}$ remains quite uncertain at between 5 and 28 ppm (*6*), and some recent iron models suggest a small effect (*13*). Last, it has been suggested that an expansion of sea ice cover off Antarctica would decrease outgassing of respired CO_2_, which, based on box model calculations, could explain as much as 67 ppm of the CO_2_ change (*14*).

An important caveat with previous studies invoking a glacial increase in the efficiency of the soft-tissue biological pump is that an accurate and complete quantification of the various carbon pumps remains lacking even for the modern ocean. One reason for this is that the carbonate chemistry of seawater buffers oceanic *p*CO_2_ changes and causes slow equilibration (~1 year) with the atmosphere. Consequently, most surface waters exhibit substantial under- or oversaturation, whose effect on the interior distribution of DIC (Fig. 1) cannot be easily separated from regenerated CO_2_ in either observations or models, and a widely used approximation based on apparent oxygen utilization (AOU) is typically used to estimate respired CO_2_ (*1*, *15*, *16*). Here, we develop and apply a novel decomposition of ocean DIC to an observationally constrained Earth System Model to confirm that AOU-based estimates substantially overestimate the inventory of respired CO_2_ (*17*–*19*) and thus underestimate the importance of disequilibrium in carbon storage in both the modern and glacial ocean. Our results suggest that the inventory of C_soft_ was lower during the LGM. However, ocean biological and physical carbon storage was enhanced largely due to an increase in air-sea disequilibrium because of temperature and iron fertilization effects, while circulation and sea ice changes played smaller roles.

To quantify carbon storage, we decompose DIC (Fig. 1A) into preformed (C_pref_) and regenerated (C_reg_) components (*1*, *20*, *21*). C_pref_ is further split (Fig. 1B) into a component C_sat_ that is in solubility equilibrium with the ambient atmosphere, and a residual disequilibrium component C_dis_. C_dis_, in turn, is a balance between disequilibrium induced by physical (C_dis,phy_) and biological (C_dis,bio_) processes. At high latitudes, ignoring biological processes, poleward-moving surface waters experience heat loss and carbon gain from the atmosphere (*22*). Because of slow air-sea gas exchange of CO_2_, further hindered by sea ice, the carbon gain is incomplete, i.e., C_dis,phy_ < 0. Thus, polar waters that sink into the deep ocean are, in the absence of biology, depleted in carbon relative to equilibrium. C_dis,phy_ therefore reduces carbon storage. Biology, on the other hand, tends to increase deep ocean DIC by C_reg_, defined as carbon that has accumulated in a water parcel during its journey from the surface to the interior. Upwelling and mixing at high latitudes, particularly around the Antarctic Divergence, brings this biogenic CO_2_ close to the surface, where, again due to slow gas exchange and sea ice, carbon loss due to outgassing to the atmosphere is incomplete, causing oversaturation (C_dis,bio_ > 0). This positive C_dis,bio_ propagates into the interior (*23*) and enhances carbon storage. C_dis,bio_ thus amplifies the biological pump (*24*). We reiterate that C_dis,bio_ is biogenic CO_2_ that is (conventionally) not included in C_reg_.

We apply this decomposition to an ocean biogeochemical model [Model of Ocean Biogeochemistry and Isotopes (MOBI)–Transport Matrix Method (TMM)] driven by circulation and forcing fields from two different configurations of the University of Victoria Earth System Climate Model (UVic ESCM) representing present-day [preindustrial control (PI)] and LGM conditions (see Materials and Methods and the Supplementary Materials) (*25*). UVic ESCM was tuned to fit a variety of present-day physical and biogeochemical observations and complementary isotopes (δ^13^C, Δ^14^C, and δ^15^N) from LGM sediments (*25*). Its LGM state is characterized by colder temperatures [global mean Δ*T* = −2.5°C, consistent with −2.6°C from ice core noble gas measurements (*26*) and sea surface temperature (SST) reconstructions (*27*, *28*)]; a shallower and ~50% weaker Atlantic Meridional Overturning Circulation (AMOC) (fig. S1), which was the only configuration of the several tested to reproduce the observed δ^13^C distribution and ~600-year-older Δ^14^C deep ocean ages (fig. S1) (*9*, *11*); and enhanced soluble iron fluxes into the Southern Ocean surface, which are required to reproduce observed δ^13^C (fig. S1) and δ^15^N (fig. S2) data. For each state, MOBI-TMM was “spun-up” to equilibrium using a fixed $p{\text{CO}}_{2}^{\text{atm}}$ (277 ppm for PI and 189 ppm for LGM) and constant phosphorous and alkalinity inventories.

To accurately partition DIC into its components, we explicitly simulate preformed DIC, nutrients, and alkalinity. Physical and biological contributions are separated by carrying out a parallel set of runs with the biological terms switched off.

## RESULTS

**Fig. 2:**
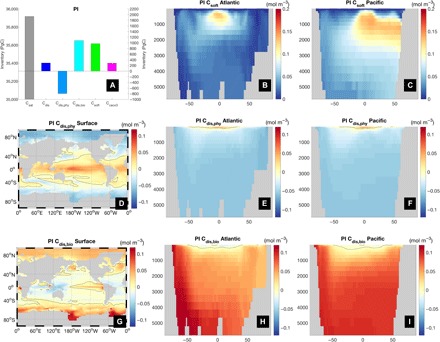
Carbon decomposition for the PI equilibrium simulation. (**A**) Inventory of preformed equilibrium carbon (C_sat_) (left axis) and other components (right axis) of DIC in the ocean. Note the different scales; (**B** and **C**) Atlantic and Pacific zonal mean meridional sections of C_soft_; (**D** to **F**) surface, and Atlantic and Pacific zonal mean C_dis,phy_; (**G** to **I**) surface, and Atlantic and Pacific zonal mean C_dis,bio_. Black solid line is the zero contour. See fig. S3 for the corresponding LGM fields.

The spatial patterns of the disequilibrium components (Fig. 2, D to I) are consistent with previous work (*22*, *23*). The total surface disequilibrium C_dis_ (fig. S3) is positive in the tropics and Southern Ocean, and negative in the subtropics, subpolar North Atlantic, and Arctic. The propagation of this C_dis_, which is higher in Southern Ocean–sourced waters than North Atlantic Deep Water, by the circulation into the ocean interior has a large impact on DIC concentrations there (*21*, *29*).

The total inventory (Fig. 2A) of C_soft_ in our PI simulation is, at 971 PgC, substantially smaller than the 1672 PgC estimated from observations (*1*) using the AOU approximation. In the latter, C_soft_ = R_C:O_(O_2,sat_ − O_2_), where R_C:O_ is a constant stoichiometric carbon-to-oxygen ratio, and O_2,sat_ and O_2_ are the temperature-dependent saturation and in situ concentrations, respectively, of dissolved oxygen. This approximation assumes that the surface O_2_ concentration is in equilibrium with the atmosphere. However, substantial disequilibrium for surface O_2_ at high latitudes during wintertime (fig. S4) propagates into the interior, leading to large errors in AOU-based C_soft_ estimation (fig. S5) (*17*–*19*), a problem that may have been exacerbated during the LGM by more extensive sea ice, consistent with other model results (*30*) and a reconstructed decrease in upper ocean oxygen concentrations there (*31*). AOU applied to our model output gives 1476 PgC, which is much closer to the data-based estimate. We thus argue that previous studies may have overestimated the inventory of respired organic carbon in the ocean by as much as 50%. As a consequence, the inventory of disequilibrium carbon, calculated as a residual between DIC and the sum of estimated C_sat_, C_soft_, and C_caco3_, may have been substantially underestimated. Our direct calculation gives a C_dis_ of 285 PgC, compared with an (AOU-based) estimate of 38 PgC (*1*). Note that C_dis_ is a balance between two large counteracting terms: a positive biological disequilibrium of +1079 PgC and a negative physical disequilibrium of −794 PgC.

### LGM versus PI carbon pumps

The lower prescribed $p{\text{CO}}_{2}^{\text{atm}}$ in the LGM run results in a smaller C_sat_ inventory (fig. S3A) despite lower temperatures that tend to increase it. There is also a ~20% decrease in C_soft_ to 795 PgC (along with concomitant decreases in C_caco3_ and regenerated PO_4_), indicating a weakening of the biological pump [as conventionally defined (*20*)]. This is consistent with the ~15% globally integrated reduction in export production (EP) simulated by the model and also seen in other simulations (*32*). The simulated pattern of change (fig. S2), in particular the “dipole” in the Southern Ocean of increased production north of the Polar Front and reduced to the south, is in good agreement with qualitative indicators (*33*). The observed increase of nitrogen isotopes in the Southern Ocean, which is affected by nutrient utilization, a variable closely linked to EP, is well reproduced by the model (fig. S2). On the other hand, using AOU (fig. S5), we obtain large increases in C_soft_ (to 1929 PgC) and regenerated PO_4_, and would (erroneously) conclude that the soft-tissue biological pump was more efficient in the LGM. This overestimate is consistent with observational evidence for oxygen depletion in near-surface waters of the glacial Southern Ocean (*31*). The decrease in C_soft_ is more than compensated by a fourfold increase in C_dis_, largely due to a doubling of the biological disequilibrium term (fig. S3A).

**Fig. 3:**
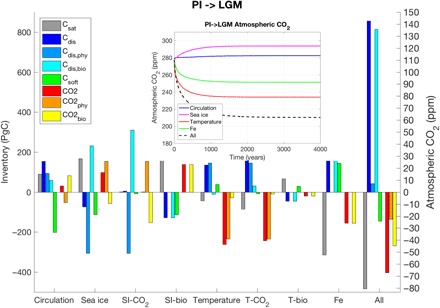
Response of PI ocean carbon cycle to LGM perturbations. Change in ocean carbon storage and atmospheric CO_2_ (inset) in response to LGM perturbations to the PI equilibrium state. (Each perturbation experiment was run for 10,000 years; only the first 4000 years are shown in the inset.) “SI-CO_2_” (“T-CO_2_”) and “SI-bio” (“T-bio”) are sensitivity experiments in which sea ice (temperature) affects only air-sea CO_2_ gas exchange or biology, respectively. The “All” experiment includes a 1-PSU increase in ocean salinity corresponding to a 110-m lowering of sea level during the LGM.

To understand the cause of these changes in carbon storage, we carried out a series of experiments with MOBI-TMM in which the PI state is perturbed, one parameter (circulation, sea ice, temperature, and iron flux) at a time. In these runs, CO_2_ is allowed to exchange freely between the ocean and a single box atmosphere such that the total amount of carbon in the combined system is conserved. In response to the perturbation, the partitioning of carbon between the ocean and atmosphere changes and evolves toward a new equilibrium state (Fig. 3), for which we diagnose the carbon components as before. Note that this experimental setup ignores changes in land and sedimentary carbon storage and whole ocean alkalinity and phosphorous, which are currently not well constrained (*34*).

### Effect of circulation changes

**Fig. 4:**
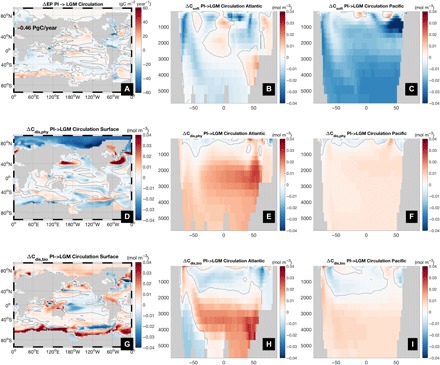
Effect of circulation changes on carbon storage. Change in (**A**) EP, (**B** and **C**) C_soft_, (**D** to **F**) C_dis,phy_, and (**G** to **I**) C_dis,bio_ due to changes in circulation. Black solid line is the zero contour.

A key finding from these sensitivity experiments is that even a large reconfiguration of circulation to a slower and shallower AMOC (fig. S1) leads to only a minor increase (~5 ppm) in $p{\text{CO}}_{2}^{\text{atm}}$. This is contrary to an extensive body of literature suggesting a large decrease in CO_2_ due to a sluggish glacial circulation (*3*, *4*, *6*–*11*, *35*). For instance, Brovkin *et al*. (*8*) attribute 43 ppm of the glacial CO_2_ decrease to AMOC shoaling, although they do not separate circulation from temperature effects in their numerical model. The modern relationship between AOU-based C_soft_ and radiocarbon age has also been applied to the LGM by converting a reconstructed radiocarbon age increase in the LGM of ~600 years to an implied increase in respired carbon concentrations (*9*, *11*) and hence lower atmospheric CO_2_ [by an estimated 67 ppm (*11*), ignoring air-sea disequilibrium]. While our model reproduces well the reconstructed pattern and magnitude of radiocarbon age increase (*11*) as a result of circulation changes (fig. S1), our arguably more accurate carbon decomposition (Figs. 3 and 4, B and C), which does not rely on AOU, shows a decrease in C_soft_ of 201 PgC. This is likely because a slower AMOC reduces upwelling of nutrients in the Indo-Pacific region and thus EP (by 0.46 PgC/year in our experiment; Fig. 4A), which is consistent with previous studies (*32*, *35*). The decrease in C_soft_ is compensated to some extent by an increase in C_dis_ of 154 PgC, as a weak AMOC reduces physical undersaturation in the North Atlantic (ΔC_dis,phy_ = 94 PgC; Fig. 4, D to F), while also filling the ocean with more high biologically disequilibrated water from the Southern Ocean (ΔC_dis,bio_ = 60 PgC; Fig. 4, G to I). Air-sea disequilibrium also provides an explanation for the simulated increase in radiocarbon age, even as the ideal mean age, a measure of transit time from the surface to the interior, decreases slightly. Surface waters in the Southern Ocean are highly depleted in radiocarbon, and the increased fraction of water sourced from that region will lead to higher apparent ages (figs. S5 and S6) (*36*).

Notably, in a reverse set of experiments (fig. S7) in which the LGM circulation is replaced by its PI counterpart, $p{\text{CO}}_{2}^{\text{atm}}$ also increases (by ~13 ppm). This state dependence (*30*), in which the net impact of circulation on CO_2_ depends quantitatively on the direction of change, suggests that circulation is not a robust factor in glacial-interglacial CO_2_ variations, even though its individual qualitative effects on C_dis,phy_, C_dis,bio_, and C_soft_ are robust.

### Effect of sea ice changes

**Fig. 5:**
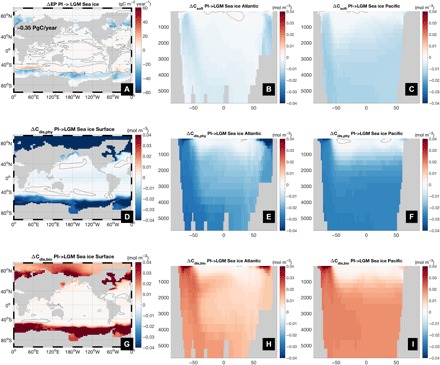
Effect of sea ice changes on carbon storage. Change in (**A**) EP, (**B** and **C**) C_soft_, (**D** to **F**) C_dis,phy_, and (**G** to **I**) C_dis,bio_ due to expanded sea ice cover. Black solid line is the zero contour.

Our LGM configuration simulates 50% more sea ice area than in PI. With a maximum cover in the Southern Ocean of 3 × 10^13^ m^2^, this is slightly smaller compared with reconstructions (4 × 10^13^ m^2^) (*37*). Replacing the PI sea ice field by the LGM one causes $p{\text{CO}}_{2}^{\text{atm}}$ to increase by 16 ppm. This contrasts with a 67-ppm decrease reported by Stephens and Keeling (*14*) based on box model simulations. Sensitivity experiments, in which the perturbed sea ice is allowed to alternatively affect only air-sea gas exchange (“SI-CO_2_”) or the penetration of light into the ocean (“SI-bio”), reveal that in the Southern Ocean the direct physical effect of sea ice on air-sea gas exchange (fig. S8) is to increase undersaturation (decreased C_dis,phy_; Fig. 5, D to F) by preventing ingassing of CO_2_ while blocking outgassing of upwelling biologically respired carbon (increased C_dis,bio_; Fig. 5, G to I) such that the net change in C_dis_ and, hence, atmospheric CO_2_ is close to zero (Fig. 3). On the other hand, sea ice blocks light, which reduces biological productivity (ΔEP = −0.26 PgC/year; Fig. 5A) (*32*, *38*), C_soft_ (Fig. 5, B and C), and C_dis,bio_ (fig. S8) (*24*), resulting in a net change in CO_2_ of +23 ppm. Neither this (*38*) nor the effect of sea ice on C_dis,phy_ was considered by Stephens and Keeling (*14*), which may explain the very large and perhaps unrealistic decrease in atmospheric CO_2_ in their model. Notably, in the reverse LGM to PI experiments, CO_2_ also increases (by ~11 ppm; fig. S7), suggesting that, like circulation, the net effect of sea ice is also state dependent and not robust.

### Effect of temperature changes

The large $p{\text{CO}}_{2}^{\text{atm}}$ decrease of 44 ppm due to cooling is the dominant effect in our model and accounts for about half of the total observed glacial-interglacial change. Sensitivity experiments similar to those for sea ice reveal that 40 ppm of this decrease can be attributed to the direct impact of temperature on solubility (“T-CO_2_”), whereas only 4 ppm result from effects on biology (“T-bio”) associated with reduced biological production, respiration, and an increase in the remineralization depth, leading to an overall increase in the respired carbon pool (fig. S9).

**Fig. 6:**
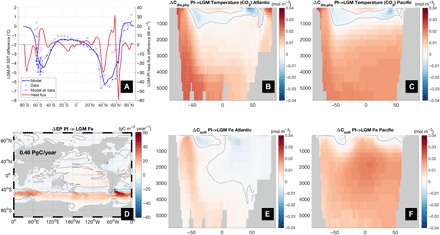
Effect of changes in temperature and iron on carbon storage. (**A**) Change in annual and zonal mean SST (left axis; solid blue line) and surface heat flux (right axis; red line) between LGM and PI. Also displayed for comparison are data from SST reconstructions [annual means from (*27*) shown as squares and summertime averages from (*28*) as circles] and model SST interpolated to data locations (dashed blue line), (**B** and **C**) Atlantic and Pacific zonal mean distribution of ΔC_dis,phy_ for the T-CO_2_ sensitivity experiment in which LGM temperature perturbations are only allowed to affect air-sea CO_2_ gas exchange, (**D**) ΔEP due to LGM iron perturbation, and (**E** and **F**) Atlantic and Pacific zonal mean distribution of ΔC_soft_ in the iron experiment. Black solid line is the zero contour.

For oceanic DIC in equilibrium with the atmosphere, theory (*1*) predicts a $p{\text{CO}}_{2}^{\text{atm}}$ decrease of 25 ppm for the observed ~2.5°C of whole ocean cooling (*26*). [The global mean SST decrease in both reconstructions (*27*, *28*) and our model is, coincidentally, nearly identical to this value.] This equilibrium effect has been confirmed by an additional sensitivity experiment (“T-const”), in which we apply a uniform cooling of 2.5°C and obtain a lowering of 24 ppm. However, temperature changes are not uniform but show greater cooling at mid-latitudes, peaking between ~40° and 60° north and south, than at high latitudes, where SSTs are fixed at the freezing point (Fig. 6A). This pattern, which is in good agreement with SST reconstructions (*27*, *28*) and other recent climate model simulations (*39*) (which to our knowledge have not been used to study the effect of cooling on CO_2_), weakens the meridional SST gradient and thus surface heat fluxes at high latitudes in the LGM, especially in the Southern Ocean south of 60°S where surface flow is poleward. This, in turn, reduces undersaturation, which is driven by heat fluxes (Fig. 2, D to F), and increases C_dis,phy_ (by 146 PgC; Fig. 6, B and C) in Antarctic Bottom Water. Spatial variability in SST thus enhances carbon storage beyond what has been considered in previous studies that have neglected the disequilibrium effect (*1*, *3*–*7*). The temperature effect is qualitatively robust and quantitatively very consistent (45 ppm in the LGM to PI experiment; fig. S7) with respect to reversing the direction of change. The good agreement of modeled temperature changes with reconstructions provides additional confidence in this result.

### Iron fertilization

Evidence from Antarctic ice and ocean sediments suggests that the flux of soluble and bioavailable iron to the Southern Ocean during the LGM may have been more than 10 times larger than at present (*40*). Our LGM model configuration is thus forced with iron fluxes enhanced by that factor south of 35°S (*25*). This results in a substantially smaller misfit (*25*) between simulated and observed δ^13^C (fig. S1), δ^15^N, and ΔEP (fig. S2).

In response to this enhanced dust flux, the model generates a decrease in $p{\text{CO}}_{2}^{\text{atm}}$ of 26 ppm (and an increase of 39 ppm for LGM to PI), which is larger than in other models (*41*, *42*) [although similar to the 22 ppm obtained by another study (*32*)]. One possible reason for this is that reconstructions of dust flux used in previous studies may have underestimated the flux of bioavailable iron to the Southern Ocean. Not only does the enhanced iron flux increase productivity (by 0.46 PgC/year) and C_soft_ (by 145 PgC; Fig. 6, E and F), but the direct impact of this fertilization is amplified by a comparable increase of ~190 PgC in biological disequilibrium carbon (fig. S9) (*24*), thus effectively more than doubling the impact of biology on ocean carbon storage, although the relationship between ΔC_dis,bio_ and ΔC_soft_ is likely to be model and state dependent (*30*).

### Effect of all changes

Consistent with the equilibrium experiments (Fig. 2A and fig. S3A), the combined effect of perturbing all variables is a large increase in C_dis_ of ~856 PgC, with major contributions from circulation, temperature, and iron (Fig. 3). C_dis,bio_ increases almost as much, mostly due to circulation, sea ice, and iron changes, whereas overall C_dis,phy_ changes are small due to compensating effects from circulation (ΔC_dis,phy_ > 0), temperature (ΔC_dis,phy_ > 0), and sea ice (ΔC_dis,phy_ < 0). Increased air-sea disequilibrium due to sea ice and higher biological productivity driven by enhanced iron flux, partly compensated by cooler temperatures and a weaker overturning circulation, also explain the lower glacial deep ocean dissolved oxygen concentrations simulated by our model and seen in proxy-based reconstructions (fig. S10) (*43*).

## DISCUSSION

The strength of the biological pump is traditionally defined in terms of the regenerated carbon (C_reg_ = C_soft_ + C_caco3_) that has accumulated in a water parcel since it was last at the surface. The large decreases in C_soft_ (by ~145 PgC) and C_caco3_ (by ~75 PgC), caused primarily by changes in circulation and sea ice, would thus imply a weaker biological pump during the LGM. However, this does not consider the effects of biology on preformed carbon. Broadening the definition (*24*) to include those effects (Fig. 1B), the biological contribution to carbon storage (C_bio_; see the Supplementary Materials) increases by 95 PgC, twice that due to physical processes (C_phy_).

The biologically mediated increase in carbon storage leads to a 67-ppm decrease in atmospheric CO_2_ (87 ppm increase for LGM to PI; fig. S7), suggesting that the model explains more than about three quarters of the observed glacial-interglacial CO_2_ change. It is likely that changes in land and sediment carbon and whole ocean alkalinity and phosphorous, which were not considered in our study, also affected glacial ocean carbon storage and atmospheric CO_2_. Future studies should focus on improved quantification of these effects without which the solution of the glacial-interglacial CO_2_ problem will remain incomplete. The model-based estimates of carbon cycle changes presented here are most likely affected by remaining model biases and uncertainties in circulation, sea ice, and other variables. Quantifying and reducing these uncertainties will be valuable objectives for future work.

We conclude that despite important contributions to individual carbon components, circulation and sea ice changes had only a modest and unrobust net effect on glacial ocean carbon storage and atmospheric CO_2_, whereas temperature and iron were more important than previously thought due to their effects on disequilibrium carbon storage. Spatial variations in temperature increase C_dis,phy_ by reducing undersaturation and thus amplify the impact of overall cooler temperatures, which accounts for about half of the total glacial-interglacial CO_2_ change. This may explain the tight coupling of CO_2_ with Antarctic temperatures observed in ice cores (*44*).

## MATERIALS AND METHODS

MOBI is a biogeochemical model with dissolved nitrogen, phosphorous, and iron as limiting nutrients; two phytoplankton functional groups; one zooplankton class; dissolved and particulate organic matter; DIC, O_2_, and alkalinity (*16*); and a prognostic iron cycle externally driven by inputs from atmospheric dust, sediments, and hydrothermal vents. Carbon and nitrogen isotopes are tracked through all the model components. MOBI is coupled to the TMM, a computationally efficient framework for offline tracer simulations (see the Supplementary Materials for additional details).

MOBI-TMM is driven by circulation, temperature, salinity, sea ice, and surface winds from UVic ESCM, a three-dimensional ocean general circulation model (1.8° × 3.6° × 19 layers) coupled to a dynamic-thermodynamic sea ice, one-layer atmospheric energy-moisture balance, and land surface models. We used PI and LGM simulations as described in (*25*). Briefly, the LGM simulation was forced with orbital parameters and atmospheric CO_2_ corresponding to 19 ka BP, a present-day climatological wind stress field to which a multimodel mean LGM anomaly from the Paleoclimate Model Intercomparison Project Phase 3 (PMIP3) was added, a continental ice sheet reconstruction from PMIP3, and a global 1-PSU (practical salinity unit) addition to salinity to account for the sea level drop. Figure S1 shows the meridional overturning circulation in the PI and LGM simulations. A detailed comparison by Muglia *et al*. (*25*) of the equilibrium solutions with observations shows consistency of simulated large-scale tracer distributions for temperature, salinity, PO_4_, NO_3_, dissolved iron, DIC, dissolved O_2_, ^14^C, δ^13^C, and δ^15^N (figs. S1 and S2).

Preformed tracers were simulated by propagating MOBI’s instantaneous, annually repeating surface fields of DIC, C_sat_, C_dis_ ≡ DIC − C_sat_, PO_4_, O_2_, and alkalinity (A_T_) as conservative tracers into the interior with the TMM. We then diagnose C_soft_ = R_C:P_(PO_4_ − PO_4,pre_) and C_caco3_ = 0.5(*p*A_T_ − *p*A_T,pre_), where R_C:P_ is a constant carbon-to-phosphorus stoichiometric ratio and potential alkalinity *p*A_T_ = A_T_ + 16 PO_4_.

We decompose DIC into physical (C_phy_) and biological (C_bio_) components such that DIC = C_phy_ + C_bio_. The physical component is defined as C_phy_ ≡ C_sat,phy_ + C_dis,phy_, and the biological one as C_bio_ ≡ C_sat,bio_ + C_dis,bio_ + C_soft_ + C_caco3_. (C_sat,bio_ is the biological contribution to equilibrium carbon. Formation of calcium carbonate shells removes alkalinity from the surface ocean, which lowers the equilibrium concentration of DIC from the value it would have in the absence of biology. C_sat,bio_ is thus generally negative. In the PI simulation, C_sat,bio_ = −292 PgC, compared with C_sat,phy_ = 36,209 PgC.)

## Supplementary Material

http://advances.sciencemag.org/cgi/content/full/5/6/eaaw4981/DC1

Download PDF

## SUPPLEMENTARY MATERIALS

Supplementary material for this article is available at http://advances.sciencemag.org/cgi/content/full/5/6/eaaw4981/DC1 Supplementary Methods Comparison of surface carbon and oxygen with observations Fig. S1. Circulation and carbon isotope distribution in the PI and LGM simulations. Fig. S2. Nitrogen isotope distribution and EP in the LGM. Fig. S3. Carbon decomposition for the LGM equilibrium simulation. Fig. S4. Comparison of simulated and observed air-sea disequilibrium. Fig. S5. Simulated AOU and radiocarbon age. Fig. S6. Effect of circulation changes on radiocarbon (Γ_C_) and ideal mean (Γ) age. Fig. S7. Response of LGM ocean carbon cycle to PI perturbations. Fig. S8. Physical and biological impacts of sea ice changes on carbon storage. Fig. S9. Effect of temperature and iron changes on carbon storage. Fig. S10. Change in dissolved oxygen concentration (ΔO_2_) in the PI-to-LGM perturbation experiments. References (*45*–*67*)
