# Supplementary material for: Air-sea disequilibrium enhances ocean carbon storage during glacial periods
Source: Sci Adv. 2019 Jun 12;5(6):eaaw4981. doi: 10.1126/sciadv.aaw4981 (PMC6561735; doi:10.1126/sciadv.aaw4981)
Supplement: http://advances.sciencemag.org/cgi/content/full/5/6/eaaw4981/DC1 [file supp_5_6_eaaw4981__index.html]

Science Advances | Science Advances

## Supplementary Materials

**This PDF file includes:**

- Supplementary Methods
- Comparison of surface carbon and oxygen with observations
- Fig. S1. Circulation and carbon isotope distribution in the PI and LGM simulations.
- Fig. S2. Nitrogen isotope distribution and EP in the LGM.
- Fig. S3. Carbon decomposition for the LGM equilibrium simulation.
- Fig. S4. Comparison of simulated and observed air-sea disequilibrium.
- Fig. S5. Simulated AOU and radiocarbon age.
- Fig. S6. Effect of circulation changes on radiocarbon (ΓC) and ideal mean (Γ) age.
- Fig. S7. Response of LGM ocean carbon cycle to PI perturbations.
- Fig. S8. Physical and biological impacts of sea ice changes on carbon storage.
- Fig. S9. Effect of temperature and iron changes on carbon storage.
- Fig. S10. Change in dissolved oxygen concentration (ΔO2) in the PI-to-LGM perturbation experiments.
- References (*45*–*67*)

Download PDF

**Files in this Data Supplement:**

- Adobe PDF - aaw4981\_SM.pdf
