# Supplementary material for: Air-sea disequilibrium enhances ocean carbon storage during glacial periods
Source: Sci Adv. 2019 Jun 12;5(6):eaaw4981. doi: 10.1126/sciadv.aaw4981 (PMC6561735; doi:10.1126/sciadv.aaw4981)
Supplement: Download PDF [file aaw4981_SM.pdf]

Science Advances
AAAS

advances.sciencemag.org/cgi/content/full/5/6/eaaw4981/DC1

Supplementary Materials for

Air-sea disequilibrium enhances ocean carbon storage during glacial periods

S. Khatiwala*, A. Schmittner, J. Muglia

*Corresponding author. Email: samar.khatiwala@earth.ox.ac.uk

Published 12 June 2019, *Sci. Adv.* **5**, eaaw4981 (2019)
DOI: 10.1126/sciadv.aaw4981

This PDF file includes:

Supplementary Methods
Comparison of surface carbon and oxygen with observations
Fig. S1. Circulation and carbon isotope distribution in the PI and LGM simulations.
Fig. S2. Nitrogen isotope distribution and EP in the LGM.
Fig. S3. Carbon decomposition for the LGM equilibrium simulation.
Fig. S4. Comparison of simulated and observed air-sea disequilibrium.
Fig. S5. Simulated AOU and radiocarbon age.
Fig. S6. Effect of circulation changes on radiocarbon (Γ_C) and ideal mean (Γ) age.
Fig. S7. Response of LGM ocean carbon cycle to PI perturbations.
Fig. S8. Physical and biological impacts of sea ice changes on carbon storage.
Fig. S9. Effect of temperature and iron changes on carbon storage.
Fig. S10. Change in dissolved oxygen concentration (ΔO_2) in the PI-to-LGM perturbation experiments.
References (*45*–*67*)

Supplementary Methods

The climate model used in our simulations is version 2.9 of UVic ESCM (*45*). Details of the PI and LGM configurations of the model are given in Refs. (*25, 46*), and the LGM continental ice sheet reconstruction from PIMP3 is described in Ref. (*47*). The iron cycle in MOBI is described in Ref. (*48*).

Biogeochemical tracers are transported using the Transport Matrix Method (TMM) (*49–51*). Briefly, the TMM is a numerical scheme for efficient simulation of ocean biogeochemical and other passive tracers. The TMM represents tracer transport due to advection, diffusion and sub-grid scale parameterizations as sparse matrices, and the time-stepping of such tracers as a sequence of sparse matrix-vector multiplications. For the MOBI-TMM offline simulations, monthly mean transport matrices (*52*) and other relevant physical forcing fields, including wind speed, insolation, sea ice concentration, temperature, salinity and freshwater flux (evaporation, precipitation and runoff), were extracted from equilibrium PI and LGM runs of UVic ESCM. All fields, including the transport matrices, were linearly interpolated to the current time step

before being applied. Equilibrium and perturbation tracer simulations were run for 10,000 years to a steady-state.

To simulate the preformed tracers, we use the annually-repeating, instantaneous surface field of the corresponding tracer from MOBI-TMM as the boundary condition, which is then advected and diffused into the interior with the TMM. A periodic (seasonally-repeating) equilibrium solution was found using a Newton-Krylov method (*53*).

Comparison of surface carbon and oxygen with observations

To assess whether there are any systematic biases in the models' ability to simulate air-sea disequlibria in O_2 and CO_2 , we have carried out a comparison of those fields with available observations. For O_2, we combined data from World Ocean Atlas (WOA) 2013 (*54*), the University of Washington Argo oxygen renalysis (*55*), and quality controlled data from floats deployed as part of the SOCCOM program (SOCCOMviz data portal (*56*); https://soccom.princeton.edu/). Data were binned spatially into the WOA $1^{\circ}\times 1^{\circ}$grid boxes, and temporally by month, to generate a seasonal climatology. Grid boxes without any measurements were discarded. Results are given in Fig. S4 (top three rows). Evidently, where there are data the model is consistent with them and suggest significant wintertime (June-July-August) undersaturation, but data in that season at high latitudes remain sparse. Other analysis (*57*) of SOCCOM float observations also show oxygen is undersaturated by up to 20% in the Seasonal Ice Zone (SIZ), in very good agreement with our model results. This broadly supports our conclusion that AOU may substantially overestimate the inventory of C_{soft} in the ocean. The largest discrepancy with observations is during summer (December-January-February), when the model does not capture the high oxygen concentrations due to photosynthesis along the edge of the SIZ.

Comparison of surface pCO_2 with data is complicated by the fact that modern measurements are contaminated by anthropogenic CO_2 and have a strong temporal trend. To address this, we

forced MOBI-TMM with observed historical atmospheric CO_2 from 1765–2018, keeping the circulation and all other physical fields fixed at their PI value, and compared simulated pCO_2 at the time and locations at which observations are available. For the data we combined monthly gridded track data from the Surface Ocean CO_2 Atlas (SOCAT), version 6 (*58*) and SOCCOM float data (*59*). As shown in Fig. S4 (bottom three rows), C_{dis}, expressed as the sea-air difference in pCO_2, is broadly consistent with existing observations (blue lines in bottom panels). Also shown are zonal averages of the PI model (green) using all grid points (dashed) and only grid points where observations are available (solid). The differences between the blue and green lines thus indicate effects of anthropogenic carbon uptake, which tends to decrease ΔpCO_2 relative to the PI. The model reproduces well the observed seasonal cycle in the subtropics, from near neutral in the summer to negative (ingassing) in winter. Also well reproduced are perennial positive (outgassing) values along the equator and large negative values in the North Atlantic and Arctic in the summer. The largest model-data discrepancy is in the high southern latitudes in summer, where the observations indicate large negative values indicative of stable stratification and biological carbon drawdown in the Ross Sea. Note that these values are unlikely to be transferred to the interior because of the stable stratification. Unfortunately, winter values at high southern latitudes, which are more likely to be subducted into the interior, are not available and therefore this important region cannot be fully evaluated. However, where there are winter data the model seems to if anything underestimate somewhat the disequilibrium. A recent analysis of SOCCOM float observations found significant outgassing (*60*), consistent with our model results of a positive disequilibrium.

We conclude that the model is broadly consistent with existing measurements of surface pCO_2 and oxygen, and that there is no evidence that the model systematically overestimates disequilibrium. However, it should be emphasized that much more data is needed to better constrain oxygen and carbon disequilibrium, especially in newly-formed dense waters.

Supplementary figures

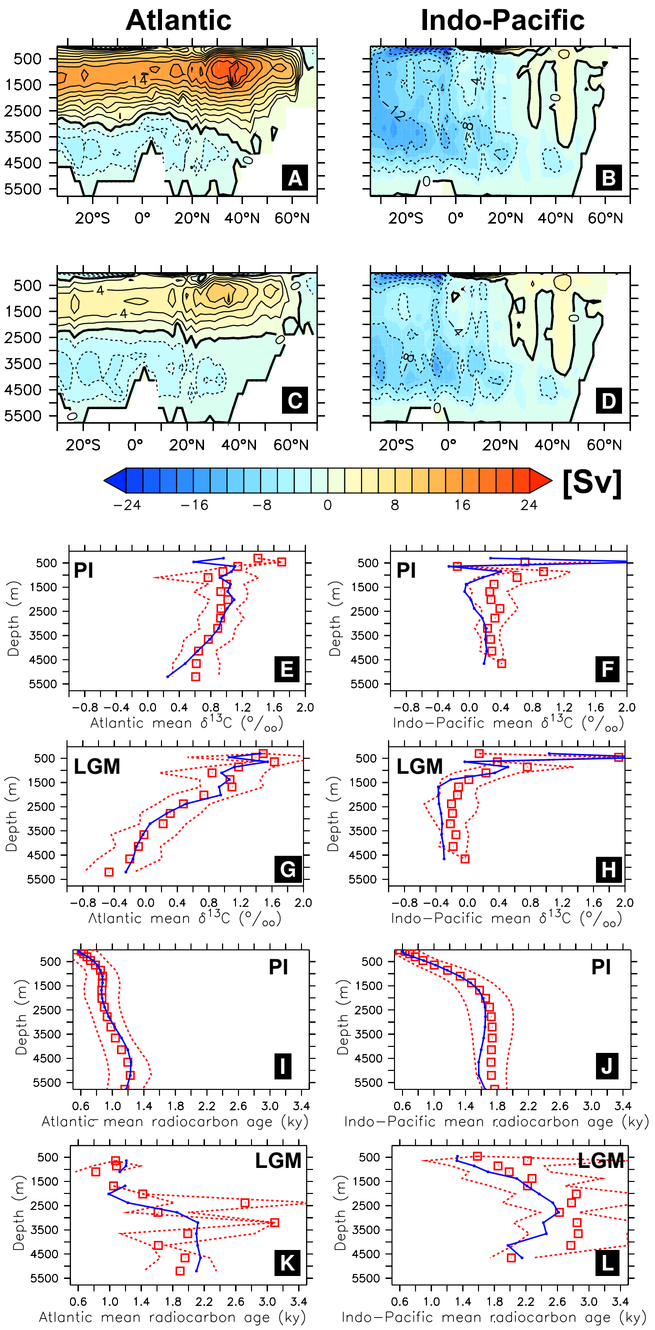

Fig. S1**. Circulation and carbon isotope distribution in the PI and LGM simulations.** Top two rows: residual meridional overturning circulation in the preindustrial control (A,B) and LGM (C,D) simulations for the Atlantic (left) and Indo-Pacific (right). Middle two rows: Atlantic (left) and Indo-Pacific (right) basin-averaged profiles of simulated (blue line) and observed (red squares) $\delta^{13}C$ for the preindustrial (E,F) and LGM (G,H). Bottom two rows: Atlantic (left) and Indo-Pacific (right) basin-averaged profiles of simulated (blue line) and observed (red squares) $\Delta^{14}C$ for the preindustrial (I,J) and LGM (K,L). Red dashed lines in the $\delta^{13}C$ and $\Delta^{14}C$ plots are the standard deviation of the data at each depth. Preindustrial $\delta^{13}C$ data are based on compilations of sedimentary core top measurements (*16, 61*), while $\Delta^{14}C$ data are water column measurements from the GLODAP database (*62*). For the LGM, $\delta^{13}C$ data are based on compilations of sedimentary core measurements (*16, 25, 61*), and $\Delta^{14}C$ data are from a compilation of sedimentary reconstrctions (*11*). Model fields were interpolated to the location of the data before being averaged.

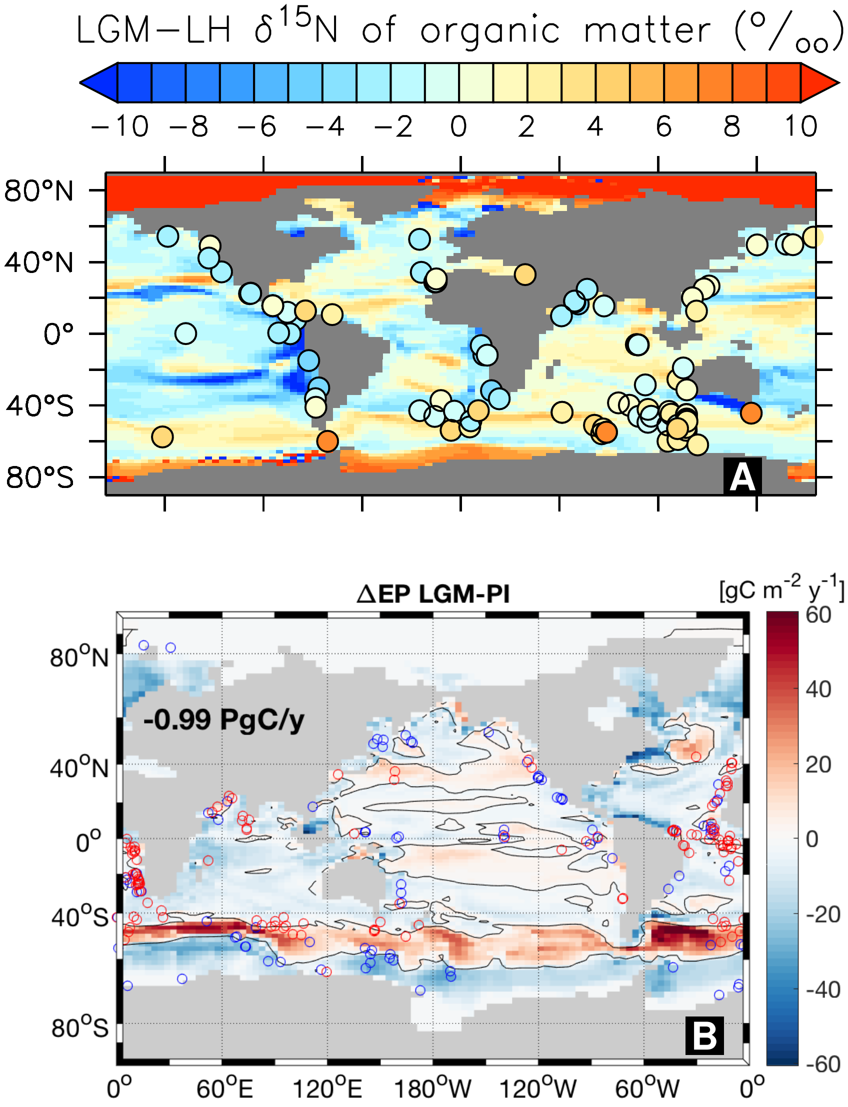

Fig. S2. **Nitrogen isotope distribution and EP in the LGM.** Top: Difference in δ^{15}N of detrital (particulate organic) matter between LGM and PI, averaged over the top 120 m of the water column. Overlaid on the plots are LGM-PI values from observations (*16, 63, 64*). Bottom: Difference in simulated export production between LGM and PI. Black solid line is the zero contour. The simulated pattern of change agrees reasonably well with proxy-based reconstructions (*33*) (circles) of the direction of change (red: LGM higher; blue: LGM lower).

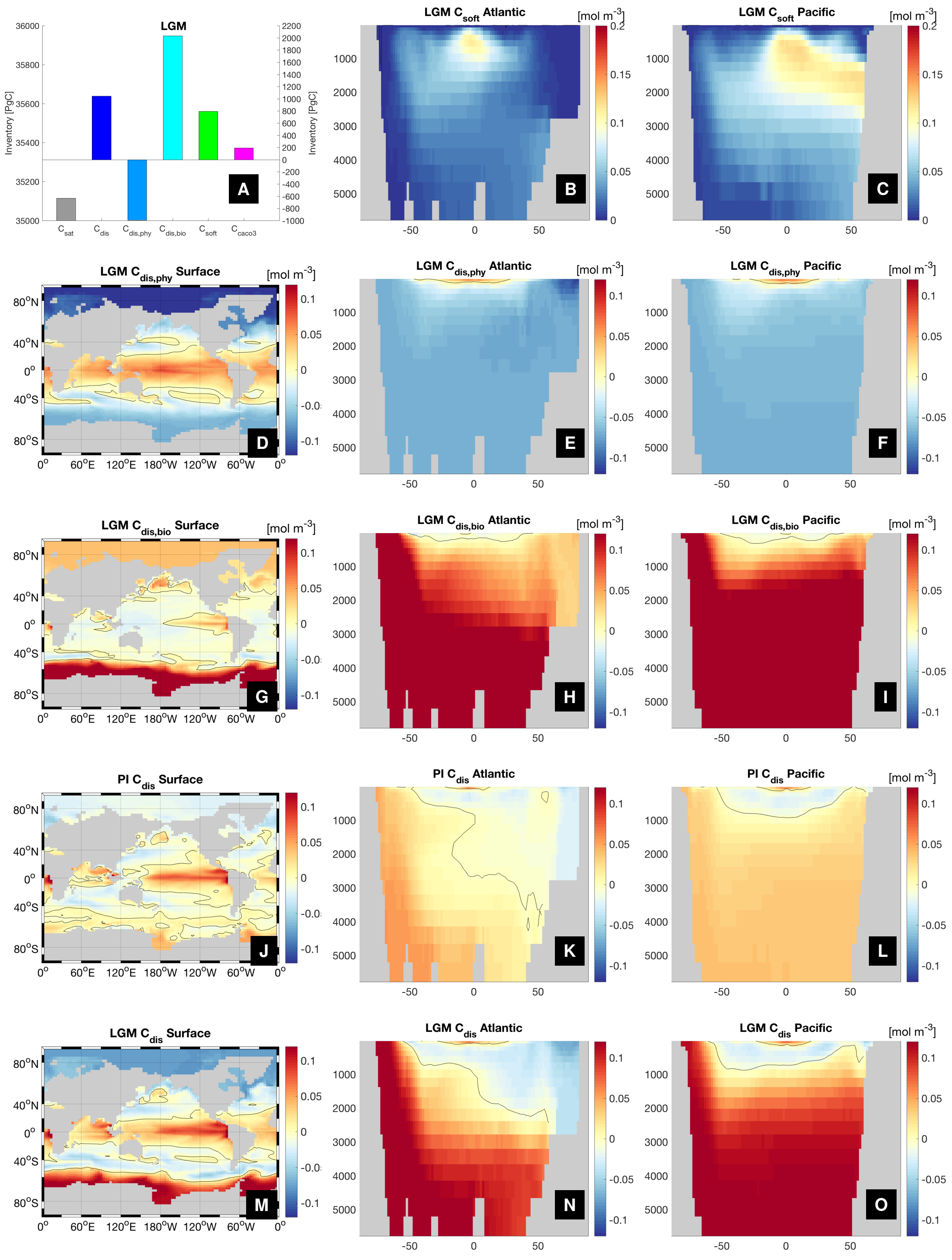

Fig. S3. **Carbon decomposition for the LGM equilibrium simulation.** (A) inventory of preformed equilibrium carbon (C_{sat}) (left axis) and other components (right axis) of DIC in the ocean. Note the different scales. The lower prescribed value of atmospheric CO_2 results in a smaller C_{sat} inventory than in PI; (B,C) Atlantic and Pacific zonal mean meridional sections of C_{soft}; (D-F) surface and Atlantic and Pacific zonal mean $C_{dis,phy}$; (G-I) surface and Atlantic and Pacific zonal mean $C_{dis,bio}$; (J-L) PI and (M-O) LGM simulated distributions of total disequilibrium carbon ($C_{dis} = C_{dis,phy} + C_{dis,bio}$) at the surface (J,M), and in the Atlantic (K,N) and Pacific (L,O). Black solid line is the zero contour.

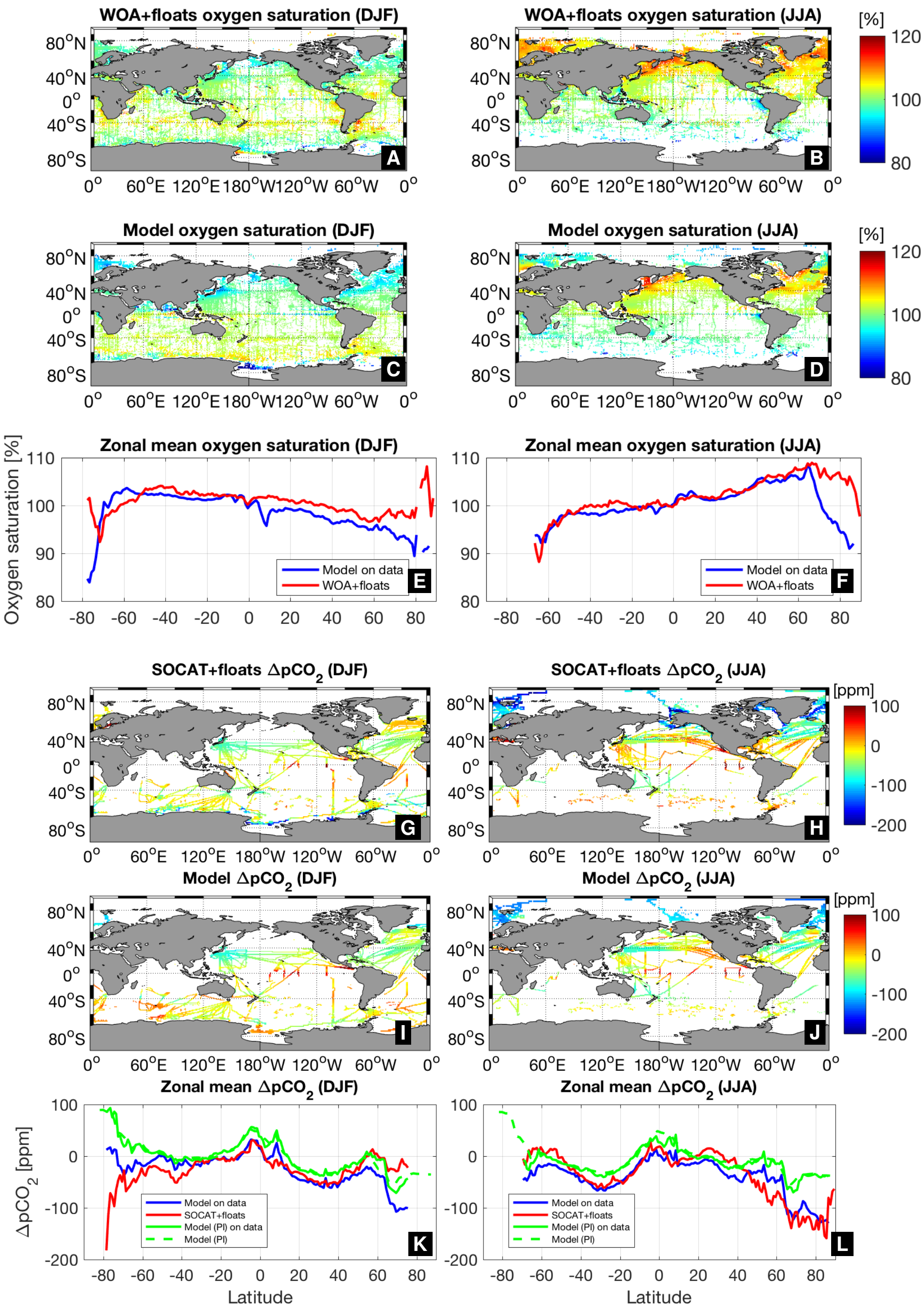

Fig. S4. **Comparison of simulated and observed air-sea disequilibrium.** Top three rows: Comparison of surface oxygen saturation in the model with observations from WOA and floats. Panels (A) and (B) show the distribution of samples in the combined data set for southern hemisphere summer (DJF) and winter (JJA), respectively. (C) and (D) show the corresponding model oxygen fields interpolated to the data locations. Lastly, the data and model fields were zonally averaged to produce panels (E) and (F). Bottom three rows: Corresponding comparison of surface pCO_2 with observations from SOCAT and floats. Panels (G) and (H) show the distribution of samples in the combined data set for southern hemisphere summer and winter, respectively; (I) and (J) the model pCO_2 fields interpolated to the time and space locations of the data; and (E) and (F) the zonally-averaged data and model fields. See supplementary text above for details of the data used and a discussion of the results.

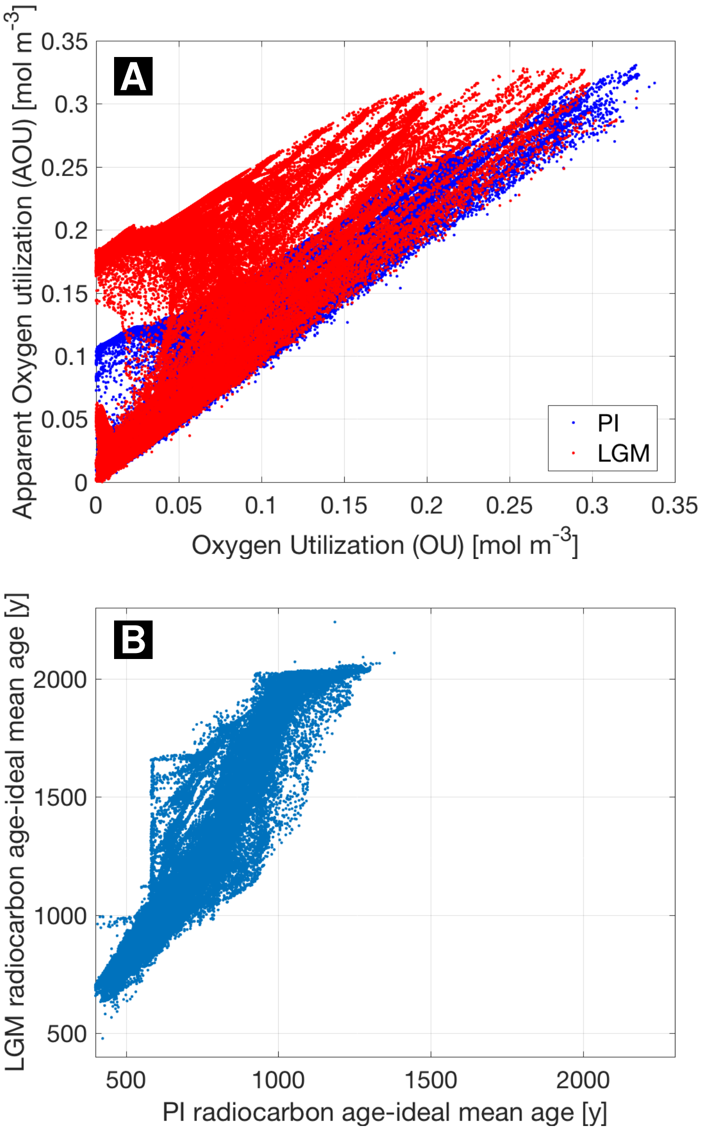

Fig. S5. **Simulated AOU and radiocarbon age.** (A) Comparison of true oxygen utilization (OU) and apparent oxygen utilization (AOU) in the PI and LGM equilibrium experiments. OU is the difference between the preformed O_2 concentration (surfaceO_2 transported passively into the interior by the circulation) and *in situ* concentration. AOU is defined as the saturation O_2 concentration, calculated at the local temperature and salinity, minus the *in situ* concentration. Data are plotted for all model grid points. AOU values are systematically higher than the corresponding OU values (*17–19*), which will translate into an erroneously higher estimate of respired carbon using the AOU method (see main text for details). The error is much larger for the LGM than PI. (B) Plot of difference between ^{14}C and ideal mean age in the PI simulation versus that in the LGM run. Data are plotted for all model grid points. Conventional radiocarbon ages are generally higher than ideal mean ages because of the reservoir age effect (*65*), but this difference increases significantly during the LGM because of the effect of circulation and sea ice on preformed ^{14}C (Fig. S6) (*36, 66*).

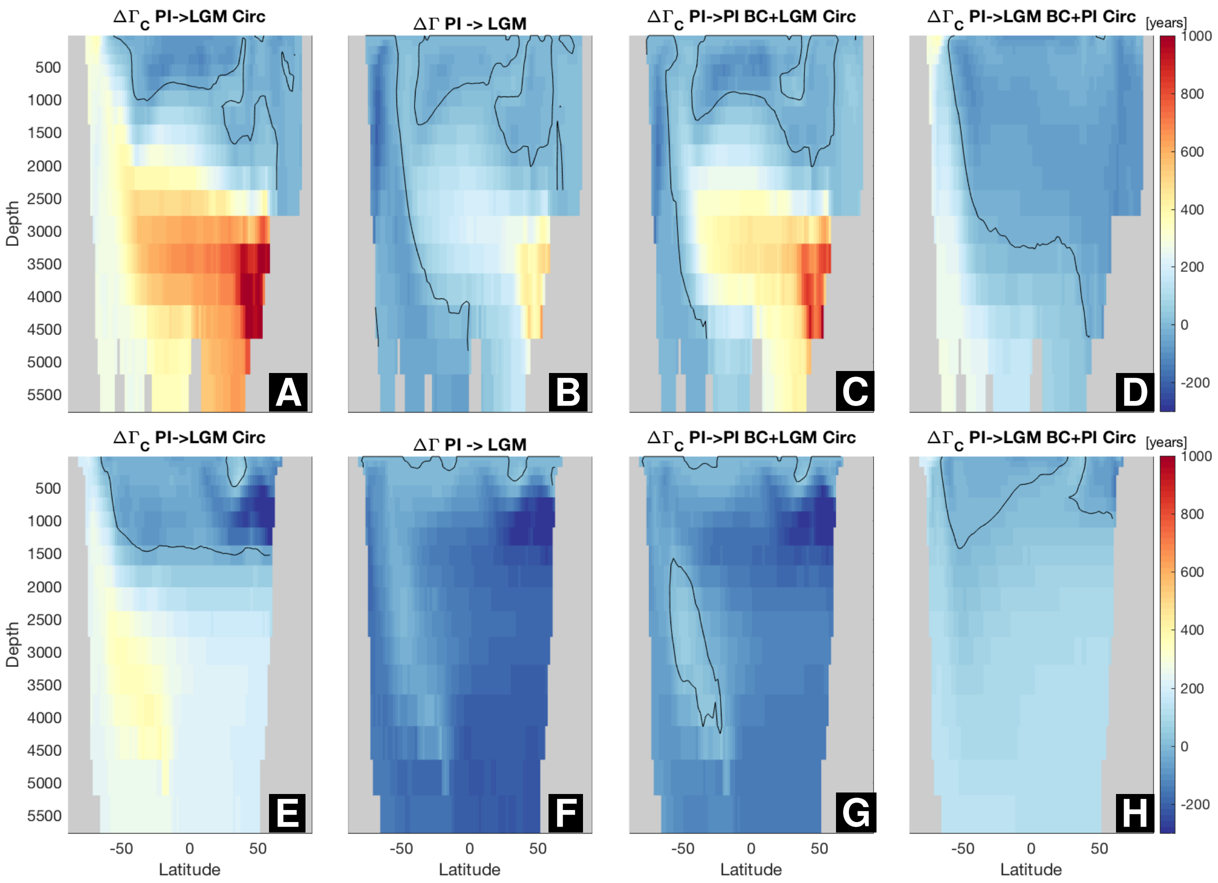

Fig. S6. **Effect of circulation changes on radiocarbon (Γ_C) and ideal mean (Γ) age.** (A,E) Change in radiocarbon age ($\Delta\Gamma_C$) in response to replacing the PI circulation with the LGM circulation. Top panels are for the Atlantic, bottom for the Pacific. Black solid line is the zero contour. Circulation not only affects how long ago a water parcel was last in contact with the atmosphere, but also the (preformed) concentration of the parcel when it was at the surface (*36, 65*), i.e., the "reservoir age". The ideal mean age on the other hand depends only on transit time and thus (B,F) the magnitude and pattern of $\Delta\Gamma$ resulting from a more Southern Ocean-ventilated circulation during the LGM are different from those of $\Delta\Gamma_C$, with much of the Pacific and bottom waters in the Atlantic experiencing shorter average transit times (*67*). This is evident from (C,G), which shows $\Delta\Gamma_C$ resulting from propagating the PI surface ^{14}C field into the interior as a radioactively-decaying tracer with the LGM circulation using the TMM. The pattern is now much more similar to that of $\Delta\Gamma$. Panels (D,H) illustrate the impact on Γ_C due to the surface boundary condition by propagating the LGM surface ^{14}C field into the interior with the PI circulation. The increase in surface reservoir age due to an altered circulation leads to apparently older bottom waters. Sea ice has a similar effect.

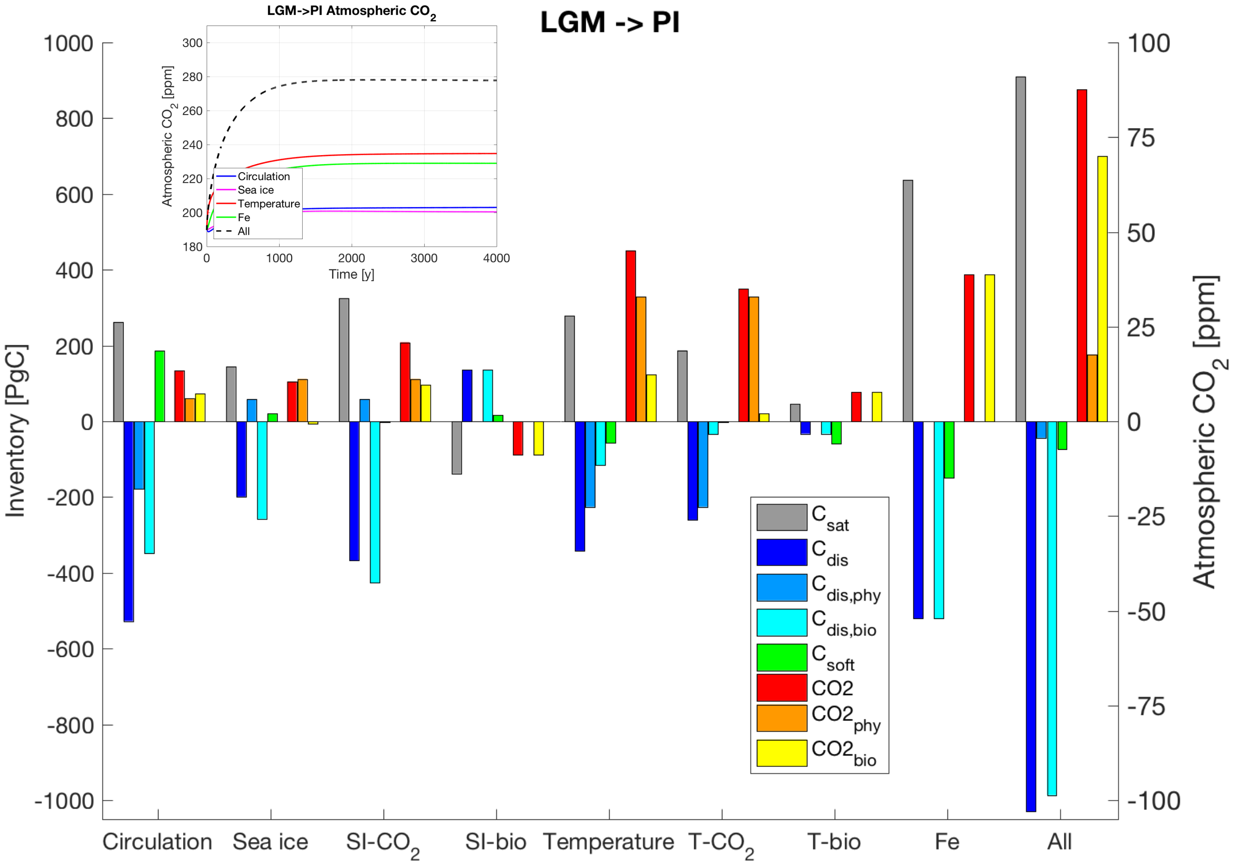

Fig. S7**. Response of LGM ocean carbon cycle to PI perturbations.** Change in ocean carbon storage and atmospheric CO_2 (inset) in response to PI perturbations to the LGM equilibrium state.

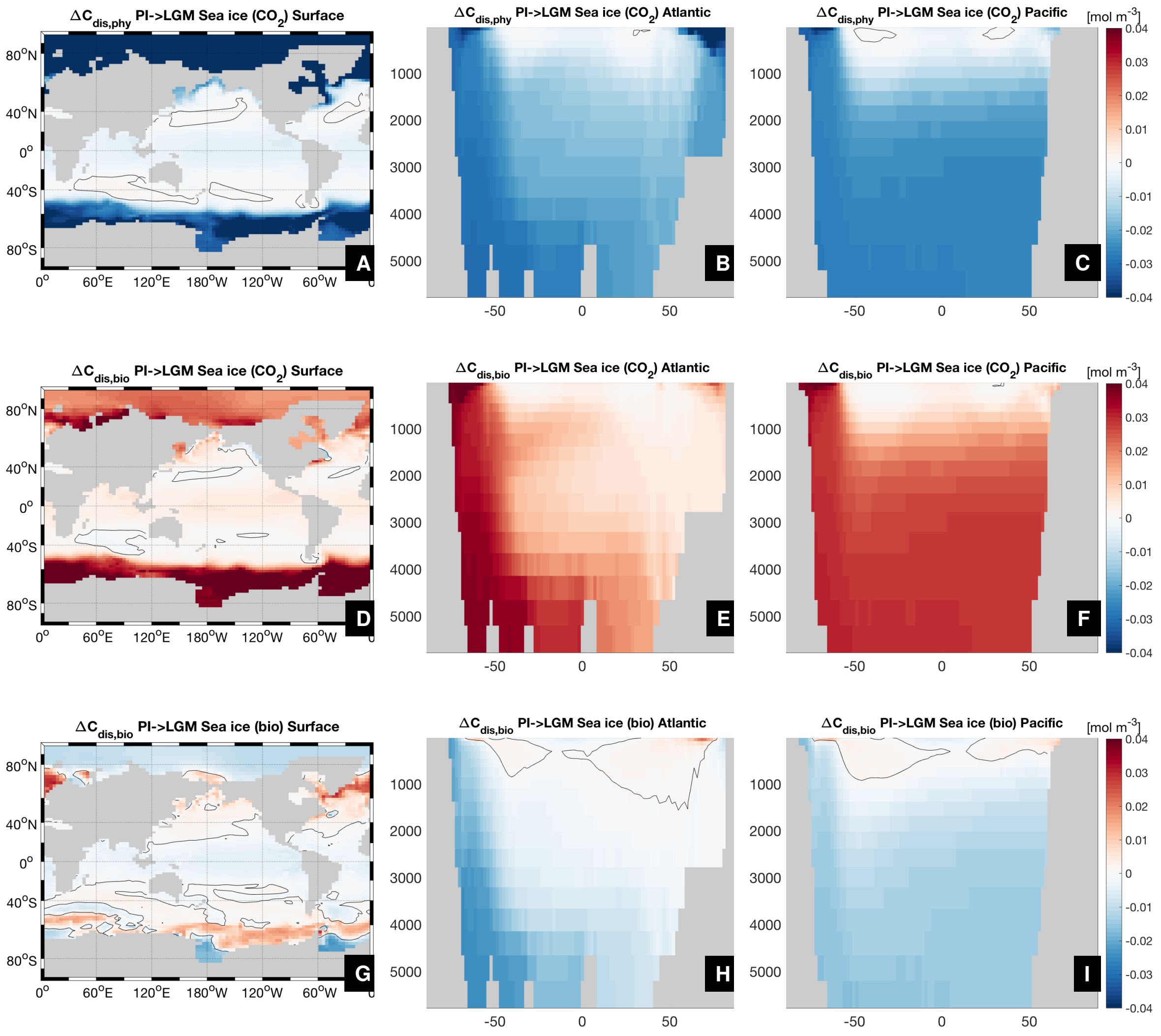

Fig. S8. **Physical and biological impacts of sea ice changes on carbon storage.** Top two rows: (A,D) Surface, (B,E) Atlantic zonal mean and (C,F) Pacific zonal mean distributions of $\Delta C_{dis,phy}$ (top) and $\Delta C_{dis,bio}$ (bottom) in the "SI-CO_2" sensitivity experiment in which the perturbed sea ice only affects air-sea gas exchange of CO_2. More sea ice prevents equilibration with the atmosphere, thus decreasing $C_{dis,phy}$ and increasing $C_{dis,bio}$. Bottom row: (G) Surface, (H) Atlantic zonal mean and (I) Pacific zonal mean distributions of $\Delta C_{dis,bio}$ in the "SI-bio" sensitivity experiment in which the perturbed sea ice only affects light penetration. Reduced export production leads to less respired CO_2 and, thence, a decrease in $C_{dis,bio}$ (*24*). Black solid line is the zero contour.

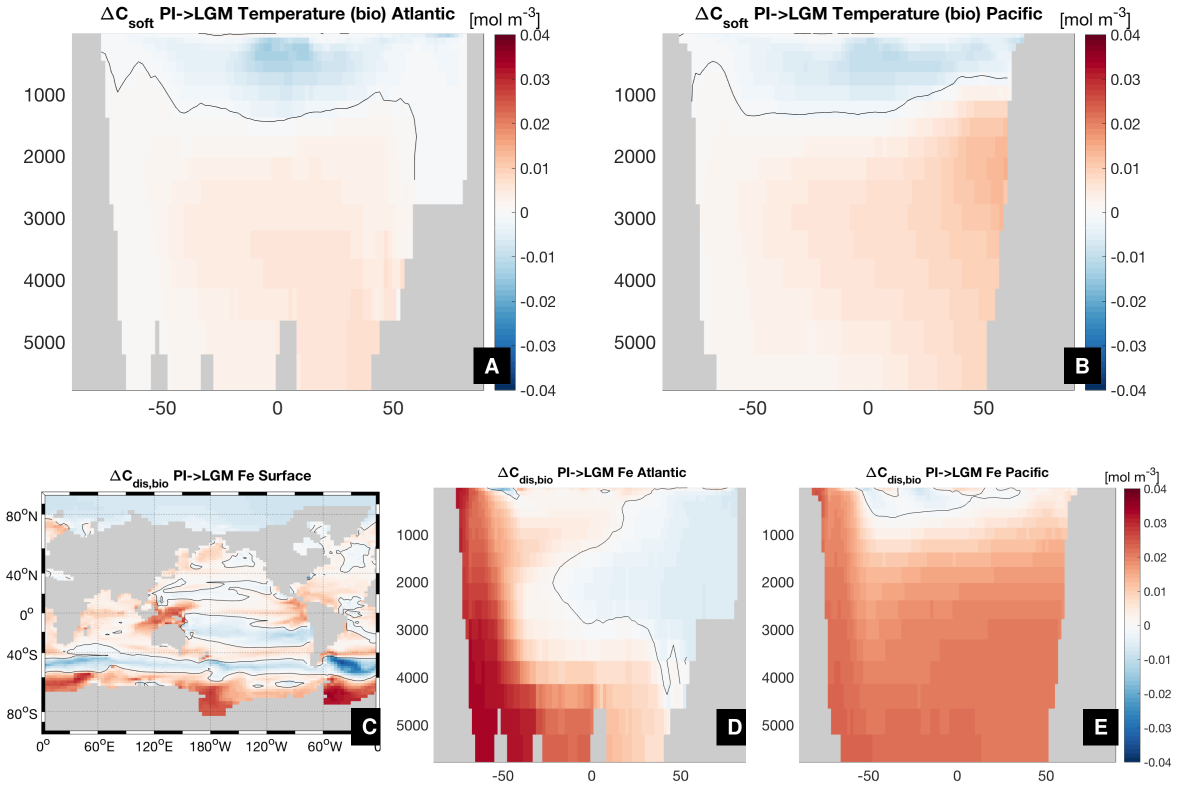

Fig. S9. **Effect of temperature and iron changes on carbon storage.** Top row: Zonal mean distribution of the change in C_{soft} in the (A) Atlantic and (B) Pacific due to effect of temperature perturbation on biology. In this "T-bio" sensitivity experiment, the PI temperature field is replaced by the LGM one *only* when it affects biology. In MOBI, phytoplankton growth and respiration rate depend on temperature. Cooler glacial temperatures reduce both, with the former leading to less export production and the latter to a deeper remineralization depth and hence increased residence time of C_{soft}. The net effect is an overall increase in the inventory of respired carbon. Bottom row: (C) Surface, (D) Atlantic zonal mean and (E) Pacific zonal mean distributions of $C_{dis,bio}$ in the iron sensitivity experiment. Black solid line is the zero contour.

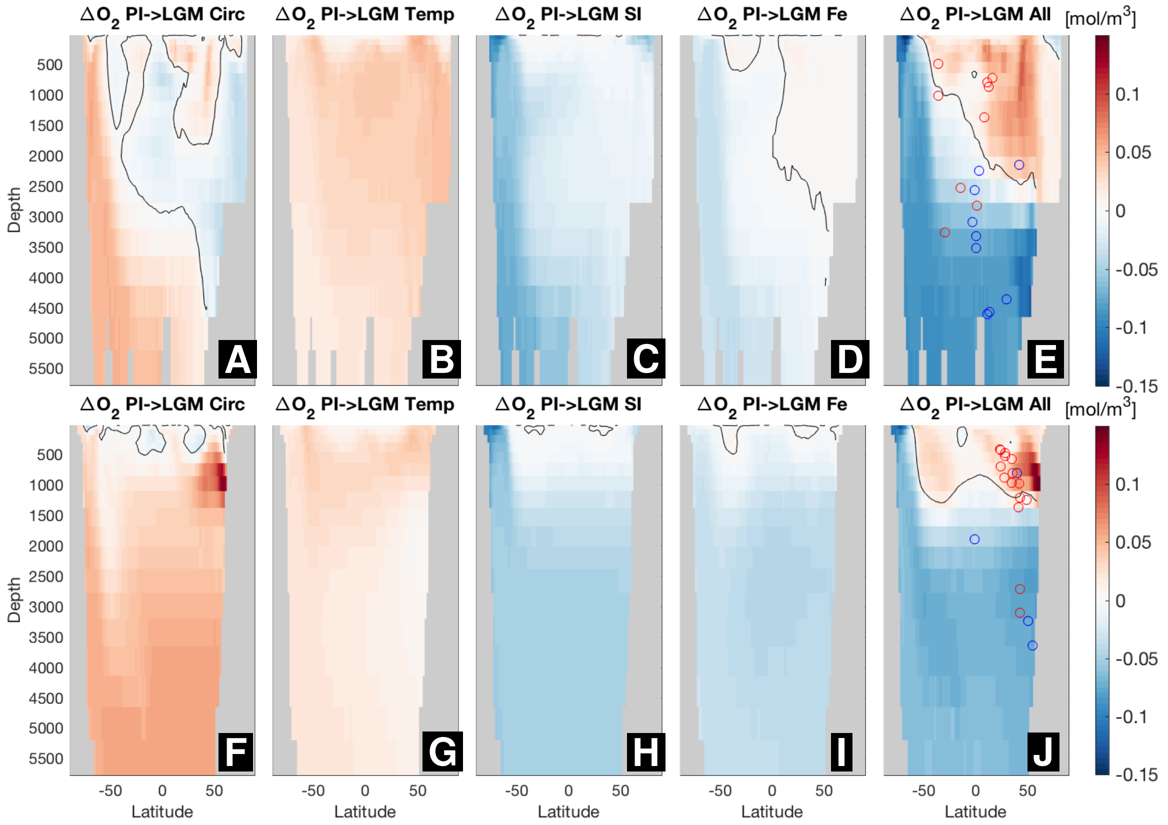

Fig. S10**. Change in dissolved oxygen concentration (**ΔO_2**) in the PI-to-LGM perturbation experiments.** (A,F) A weaker AMOC causes both less export production (Fig. 4) and shorter transit times (Fig. S6), leading to higher deep ocean O_2 concentrations. Top panels are for the Atlantic, bottom for the Pacific. Black solid line is the zero contour. This is compounded by the effect of cooler temperatures (B,G), but compensated by air-sea disequilibrium due to sea ice (C,H) and higher biological productivity driven by enhanced iron fluxes (D,I) during the LGM. The net effect (E,J) of simultaneously perturbing all parameters is to decrease O_2 concentrations in the deep ocean and increase them in the upper ocean during the LGM. This simulated pattern of change agrees reasonable well with with proxy-based reconstructions (*43*) (circles in panels E and J) of the direction of change (red: LGM higher O_2; blue: LGM lower).
